# The benefit of using the AAPM Journal app

**DOI:** 10.1002/acm2.13019

**Published:** 2020-08-30

**Authors:** Amy S. Yu

**Affiliations:** ^1^ Stanford Radiation Oncology Palo Alto CA USA

## 1 The History of the *AAPM Journals* app

The way we receive information has changed. Twenty years ago, if you wanted to make an announcement of your graduation or celebrate a job promotion, you called your friends or wrote them letters and a small portion of them would get the good news. Today, you only need to send out an email or share the news on social media and all of your friends would know instantly. A Journal Editorial Board Working Group was formed with the aim to improve the reader experience by enhancing accessibility and readability of our journals in this new era with the lead and guidance from Dr. Colin Orton. The members of the Working Group are exploring how to provide a way for medical physicists to quickly and easily access the published articles. With Wiley Publishing’s help, a smart device app, *AAPM Journals*, was developed to not only provide access to published articles from both Medical Physics and JACMP but also to be easy to use. The *AAPM Journals* app launched in 2018 and is available for free on Android, iPhone, and iPad. We will discuss the benefits and functions of this app for medical physicists in this article.

## 2 Why should you use the AAPM Journals app?

As a medical physicist, you want to stay up to date on the latest innovations and studies in our field. Technology has influenced how and where this information is accessed and shared. In the past, we waited for the hardcopy of the journal to arrive in the mail and would go through it to see if there were any interesting articles or, more likely, the journal was placed on the bookshelf until we had time to read it. Now, we expect access to information to be convenient and in real time, so instead of waiting until an article is fully published in print, we prefer to have on‐demand access to it as soon as it is accepted. With the use of *AAPM Journals*, you can access and read articles immediately at your convenience, anytime and anywhere. You no longer need to be in front of the computer or in your office with the journal open on your desk. It is more convenient if you can pull out the studies on the go; at the treatment unit, during your commute or a mentoring session. Moreover, a major benefit is that readers can download complete issues in a single step which can only be done with *AAPM Journals*!

In order to provide a hassle‐free app, readers can simply log in with their AAPM member accounts; it does not need institutional credentials or provide any payment. There is no login requirement for JACMP since it was established as an Open Access journal in 2000 with the vision of Dr. Michael Mills. Moreover, you can share interesting articles with your co‐workers via the app, so good studies will travel faster and farther. By sharing an article, it would increase its visibility and possibly increase the number of citations. It is also easy for scientists to stay up to date and discover curated and relevant articles when they are released through push notifications. Users can create custom alerts to include chosen keywords and the app will send you notifications when new content is published allowing you to discover what matters most to you.

The functions and benefits of using the AAPM Journal app

- It is free and available for both Android and iOS.
- Login with your AAPM member account: You do not need to have institutional credentials or provide payment to the publisher.
- Bookmark articles and read later: You can discover research at your own pace.
- Download interesting articles or complete issues: You can read the articles offline, for example, you can read the article on the airplane.
- Push notifications and alerts: You can select keywords to get personalized notifications and be the first one to discover relevant content.
- Increase the visibility of your article: It is easy to share the articles with friends and collaborators.

How do I get started?

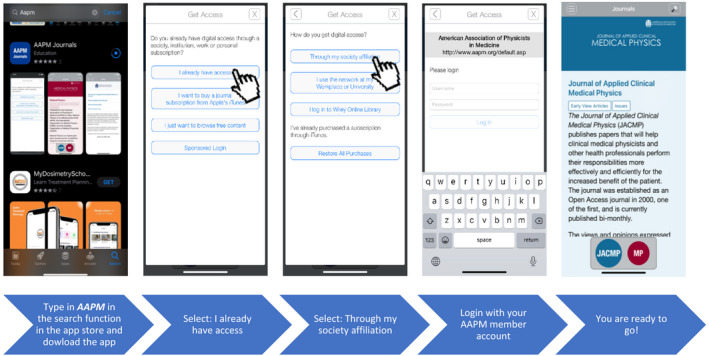

## 3 Conclusion

*AAPM Journals* provides the convenience and real‐time accessibility to allow you to read articles anytime and anywhere. We anticipate that this app will change the way we read articles and the way we receive new information. Please help us to spread the word. You can share the information with your coworkers in the laboratory or introduce it in clinical meetings and local Chapters. This will be a great way to promote your research and stay updated in this field. If you have any suggestions or questions, please email us (amysyu@stanford.edu) and we would love to hear your feedback.

